# Readmission rates in HIV-associated burkitt lymphoma patients in the USA: a nationwide readmission database (NRD) analysis

**DOI:** 10.1186/s12981-023-00575-3

**Published:** 2023-11-11

**Authors:** Ashley M. Tuin, Clare M. Wieland, Elizabeth J. Dort, Danielle B. Dilsaver, Manasa Velagapudi

**Affiliations:** 1 Creighton University School of Medicine, CHI Health, Omaha, NE USA; 2 Department of Clinical Research & Public Health, Creighton University School of Medicine, Omaha, NE USA; 3 Division of Infectious Diseases, Department of Internal Medicine, Creighton University School of Medicine, Omaha, NE 68154 USA

**Keywords:** Burkitt Lymphoma, HIV, Antiretroviral therapy, Hospital readmission, NRD, HIV associated Burkitt Lymphoma, Lymphoma

## Abstract

**Background:**

People with human immunodeficiency virus have an increased risk of developing AIDS-defining malignancies including Burkitt lymphoma. Survival outcomes in HIV-associated Burkitt lymphoma remain worse than non-HIV-associated Burkitt lymphoma, despite widespread implementation of antiretroviral therapy. We aimed to determine the association between HIV status and risk for 30-day and 90-day readmission in the US after index hospitalization for Burkitt lymphoma.

**Methods:**

Data were abstracted from the 2010–2020 Nationwide Readmissions Database; hospitalizations included patients with a primary BL diagnosis and were stratified by comorbid HIV. The primary outcome was all-cause readmission (30-day and 90-day). Secondary outcomes were in-hospital mortality, length of stay (LOS), and hospital cost. Between-HIV differences were evaluated via logistic and log-normal regression; multivariable models adjusted for comorbid kidney disease, hypertension, fluid and electrolyte disorders, and sepsis.

**Results:**

Overall, there were 8,453 hospitalizations for BL and 6.0% carried an HIV diagnosis. Of BL hospitalizations, 68.4% were readmitted within 30-days post index BL hospitalization and 6.8% carried a HIV diagnosis. HIV-associated BL was associated with 43% higher adjusted odds of 30-day readmission (aOR 95% CI: 4% higher to 97% higher, p = 0.026). For 90-day readmission, 76.0% of BL patients were readmitted and 7.0% carried a HIV diagnosis. HIV-associated BL was not statistically associated with all-cause 90-day readmission (aOR 1.46, aOR 95% CI: 0% higher to 115% higher, p = 0.053).

**Conclusions:**

HIV-positive status is associated with an increased risk for 30-day readmission after index hospitalization for Burkitt lymphoma.

## Background

Human immunodeficiency virus (HIV) remains an epidemic worldwide with an estimated 36,136 new HIV diagnoses in the US in 2021 [1, 2]. According to the CDC, 75% of HIV-positive patients receive antiretroviral therapy (ART); however, only 66% are virally suppressed [2]. Despite increased survival in the ART era [3], people with HIV (PWH) have a higher incidence and mortality of advanced-stage cancer, particularly AIDS-defining malignancies of Burkitt lymphoma (BL) [4, 5]. PWH and BL experience higher mortality rates than patients carrying a lymphoma diagnosis without an HIV diagnosis [6, 7]. Additionally, evidence shows that outcomes of HIV-associated BL are based on tumor and cancer specific factors, opposed to HIV specific factors [8, 9]. In the United States from 2015 to 2019, non-Hodgkin’s lymphoma had an incidence rate of 449.4 cases per 100,000 people. Relatedly, the American Cancer Society estimates nearly 1.95 million new cases of non-Hodgkin’s lymphoma in 2023 [10]. BL is as a rare, yet aggressive form of non-Hodgkin’s lymphoma in both the general and immunodeficient populations, thus making people with BL an important population to study [11]. Current literature acknowledges worse outcomes in PWH with BL, however rates of hospital admissions and readmissions in this group are not studied. Studying these factors can help clinicians to educate their patients of the expected outcomes. Given the increased mortality and decreased survival of HIV-associated BL, we explored the association of HIV status on readmission rates, patient demographics, and presence of comorbid conditions in patients admitted for BL in the US using data from 2010 to 2020 in the Nationwide Readmissions Database (NRD).

## Methods

### Data source

Study data were abstracted 2010–2020 NRD. Index hospitalizations included patients with a primary BL diagnosis stratified by comorbid HIV status as indicated by ICD-9/10 codes (available upon request). Hospitalizations in which the patient was younger than 18 years old were excluded.

### Outcomes

#### Study outcomes

Primary outcomes were all-cause and cause-specific 30-day and 90-day readmission rates. Cause-specific readmission rates (reason for readmission) were indicated by Medicare Severity Diagnosis Related Groups (MS-DRG). To allow for complete follow up, index hospitalizations in which the patient died were excluded. For 30-day readmissions, index hospitalizations in which the patient was discharged in December were excluded. For 90-day readmissions, index hospitalizations in which the patient was discharged in October, November, or December were excluded. Secondary outcomes included in-hospital mortality, length of stay (LOS), and hospital cost inflation-adjusted to mid-year 2020 US dollars [12].

#### Covariates

Descriptives were extracted at index hospitalization; these included age, sex, insurance (Medicaid, Medicare, private, other), income quartile (I, II, III, IV) and heart failure, kidney failure/disease, fluid/electrolyte disorders, sepsis/septic shock, and cerebrovascular accident/occlusion of precerebral arteries with infarction. Refined Elixhauser comorbidities were also identified; these included hypertension, alcohol abuse, rheumatoid arthritis/collagen vascular disorders, depression, diabetes, drug abuse, chronic pulmonary disease, obesity, peripheral vascular disorders, and hypothyroidism.

### Statistical analysis

Logistic regression models were estimated to evaluate if the odds of all-cause 30-day and 90-day readmission and inpatient mortality differed by HIV status. Cause-specific 30-day and 90-day readmission rates were presented as frequency and percentage. Log-normal models were estimated to evaluate whether LOS and hospital cost differed by HIV status. Multivariable models were estimated to control kidney disease, hypertension, fluid and electrolyte disorders, and sepsis.

Specific to HIV-associated BL hospitalizations, a logistic regression model was estimated to assess whether the odds of readmission differed by years when antiretroviral therapy was based on CD4 count and presence of co-morbidities (2010–2014) compared to years when universal antiretroviral therapy with integrase inhibitors as part of ART was a standard treatment for HIV (2015–2020) [13, 14].

## Results

In the US from 2010 to 2020, there were an estimated 8,453 (95% CI: 8,089 to 8,818) BL hospitalizations; 6.0% carried an HIV diagnosis (weighted N = 510, 95% CI: 436 to 585). HIV-associated BL hospitalizations were younger patients and had a greater proportion of men compared to hospitalizations without HIV (44 years vs. 56 years, p < 0.001; 85.2% vs. 70.6%, p < 0.001). Overall private insurance was the most common primary payer (HIV: 40.6% vs. No HIV: 43.5%, p < 0.001). HIV-associated BL hospitalizations were more frequently in the lowest income quartile compared to hospitalizations without HIV (38.2% vs. 23.6%, p < 0.001). HIV-associated BL hospitalizations had a smaller proportion of patients with kidney disease and hypertension compared to the non-HIV cohort (16.5% vs. 27.0%, p = 0.008, 9.9% vs. 17.4%, p = 0.001). All other comorbidities were not statistically associated with HIV status or their prevalence in HIV-associated BL hospitalizations was too low to report per the NRD Data Use Agreement [15] (Table 1).

**Table 1:** Demographic and clinical characteristics of HIV-associated BL hospitalizations

	HIV: No	HIV: Yes	p
Demographic Characteristics			
Age	56 (40, 68)	44 (35, 50)	< 0.001
Biological Sex			
Male	70.56	85.21	< 0.001
Female	29.44	14.79	
Insurance			
Medicare	32.36	7.69	< 0.001
Medicaid	16.73	39.71	
Private	43.45	40.58	
Other	7.43	12.03	
Income Quartile			
I	23.56	38.17	< 0.001
II	27.43	27.44	
III	26.15	20.69	
IV	22.85	13.71	
**Clinical Characteristics**			
Kidney failure and disease	27.03	16.51	0.002
Hypertension	17.39	9.92	0.001
Fluid & electrolyte disorders	40.01	38.65	0.689
Sepsis, septic shock	9.91	8.75	0.523
Depression	4.51	4.28	0.861

a. HIV = Human immunodeficiency virus. BL = Burkitt lymphoma

b. Categorical variables were presented as percent and compared using Rao-Scott chi-square test. Continuous variables were presented as median and interquartile range and compared using regression to allow for NRD weighting

c. Between-HIV differences in comorbid heart failure, cerebrovascular accident and occlusion of precerebral arteries with infarction, alcohol abuse, rheumatoid arthritis and collagen vascular disease, diabetes, drug abuse, chronic pulmonary disease, obesity, peripheral vascular disorders, and hypothyroidism could not be reported per the NRD Data Use Agreement; the number of unweighted hospitalizations was 10 or less

d. The NRD sampling design was accounted for in all analyses using SAS v. 9.4 with two-tail p < 0.05 indicating statistical significance

### Outcomes

Of index BL hospitalizations, 68.4% (weighted N = 4,756, 95% CI: 4,505-5,008) were readmitted within 30-days post index hospitalization; 6.8% carried a HIV diagnosis (weighted N = 324, 95% CI: 270–377). HIV was associated with 45% higher unadjusted odds of all-cause 30-day readmission compared to BL hospitalizations without a HIV diagnosis (75.4% vs. 67.9%, respectively; OR 95% CI: 6% higher to 99% higher, p = 0.021; Table 2). Adjusting for comorbidity burden, HIV-associated BL was associated with 43% higher adjusted odds of 30-day readmission compared to BL hospitalizations without a HIV diagnosis (aOR 95% CI: 4% higher to 97% higher, p = 0.026; Table 2). The most common reasons for 30-day readmissions were chemotherapy (weighted N = 3,887; 54.96%), lymphoma-specific (weighted N = 718; 10.15%), major hematological and immunological diagnosis (weighted N = 644; 9.11%), sepsis or severe septicemia (weighted N = 300; 4.23%), and HIV-specific (weighted N = 218; 3.08%).

**Table 2:** Primary and secondary outcomes stratified by comorbid HIV

	Unadjusted				Adjusted	
	HIV: No	HIV: Yes	Ratio (95% CI)	*p*	Ratio (95% CI)	*p*
Index Hospitalization						
In-patient Mortality (%)	10.83	7.89	0.71 (0.38–1.32)	0.272	0.81 (0.38–1.70)	0.575
Length of Stay (days)	9.23	8.80	0.95 (0.81–1.13)	0.574	1.00 (0.85–1.17)	0.960
Cost ($)	32,735	28,195	0.86 (0.76–0.98)	0.022	0.92 (0.81–1.03)	0.144
All-cause Readmission						
30-day (%)	67.90	75.42	1.45 (1.06–1.99)	0.021	1.43 (1.04–1.97)	0.026
90-day (%)	75.51	82.42	1.52 (1.04–2.23)	0.031	1.46 (1.00-2.15)	0.053

a. HIV = Human immunodeficiency virus

b. CI = Confidence Interval

c. The NRD sampling design was accounted for in all analyses using SAS v. 9.4 with two-tail p < 0.05 indicating statistical significance

For all-cause 90-day readmission, 76.0% (weighted N = 4,302, 95% CI: 4,080 − 4,523) of BL patients were readmitted within 90-days post index BL hospitalization; 7.0% carried a HIV diagnosis (weighted N = 299, 95% CI: 250–349). HIV was associated with 52% higher unadjusted odds of readmission compared to non-HIV BL hospitalizations (82.4% vs. 75.7%, respectively; OR 95% CI: 4% higher to 123% higher, p = 0.031; Table 2). However, HIV-associated BL was not statistically associated with all-cause 90-day readmission after adjusting for kidney disease, hypertension, fluid and electrolyte disorders, and sepsis, (aOR 1.46, aOR 95% CI: 0% higher to 115% higher, p = 0.053; Table 2). Similar to 30-day readmissions, the most common reasons for 90-day readmissions were chemotherapy (weighted N = 8,749; 61.46%), major hematological and immunological diagnosis (weighted N = 1,298; 9.12%), lymphoma-specific (weighted N = 1,094; 7.68%), sepsis or severe septicemia (weighted N = 550; 3.87%), and HIV-specific (weighted N = 403; 2.83%).

Specific to HIV-associated BL hospitalizations, there were an estimated 291 (95% CI: 266–316) index hospitalizations during the CD4/comorbidity-based ART era compared to 219 (95% CI: 210–228) index hospitalizations during the universal ART era. Timeframe (CD4 era vs. Universal era) was not significantly associated with all-cause 30-day and 90-day readmissions (30-day readmission: 78.3% vs. 71.9%; OR: 0.71, 95% CI: 0.43 to 1.19, p = 0.189; 90-day readmission: 85.3% vs. 78.6%; OR 0.63, 95% CI: 0.34 to 1.17, p = 0.141).

## Discussion

These results indicate a difference in readmission rates by HIV status. The current study demonstrated that in BL hospitalizations, comorbid HIV was associated with a significantly higher unadjusted and adjusted 30-day readmission rate and a significantly higher unadjusted 90-day readmission rate (p = 0.021, p = 0.026, p = 0.031, respectively). Hospitalizations of patients carrying an HIV and BL diagnosis had 43% higher odds of readmission within 30 days after discharge, after adjusting for common comorbidities (p = 0.026). However, there was no significant difference in 90-day readmission rate in HIV-positive BL patients after adjusting for comorbidities (p = 0.053). Of note, the unadjusted 30-day and 90-day readmission rates were statically significant, indicating a greater overall risk of readmission for PWH and BL.

One possible explanation for the increased readmission rate is the increased toxicity of the chemotherapy regimen in PWH. In our study, the most common reason for 30-day and 90-day readmission was chemotherapy at 54.96% and 61.46%, respectively. A 2017 study by Xiao, et al. [16], demonstrates more severe toxicity to the CODOX-M/IVAC ± R chemotherapy regimen was experienced by HIV-positive patients due to lower CD4 + counts and lower Karnofsky performance status. Difficulty in tolerating chemotherapy could require more supportive measures during treatment and therefore higher readmission rates. Another possible explanation for this increased readmission rate is the higher rate of infection during treatment. In 2021, Wang, et al. [17] demonstrated almost 60% of PWH and lymphoma experienced infections during chemotherapy. Patients with a higher number of chemotherapy cycles, grade 4 decrease in neutrophil counts, and less than 6-month duration of ART at time of diagnosis were all independent infection risk factors [17]. The combination of myelosuppression and already reduced CD4 count creates a uniquely higher risk of infection development over the course of treatment for this patient population [17]. However, our study found that rate of sepsis-related readmissions was only 4.23%for 30-day readmissions and 3.87% for90-day readmissions. We were not able to determine if other infections impacted readmissions for other hematological and immunological diagnoses due to administrative coding limitations (MS-DRG and ICD coding). Notably, readmissions for other hematological and immunological diagnoses were more common than sepsis-specific readmissions (30-day readmission: major hematological and immunological diagnosis 9.11% vs. sepsis-specific 4.23%l; 90-day readmission: major hematological and immunological diagnosis 9.12% vs. sepsis 3.87%).

BL survival outcomes increased from 10 to 40% in the pre-ART era to 70–80% in the current era, indicating advancements in antiretroviral therapy and HIV treatment may have contributed to increased survival in HIV-associated lymphomas [11]. However, a recent National Cancer Database study demonstrated lymphoma patients with HIV continue to experience worse survival even following implementation of ART, leading us to stratify readmission rates during different time periods of ART implementation in our study [6]. Our data demonstrates universal ART and use of integrase inhibitor based regimens were not associated with any significant differences in readmission rates for those with HIV-associated BL for all-cause 30-day and 90-day readmission. Our findings further support previous data indicating outcomes among HIV-associated BL patients no longer associate with HIV specific features but are more likely to be associated with cancer and treatment specific features [8, 9].

Notably, our results showed between-HIV status disparities in socioeconomic factors, particularly income quartiles. Of HIV-associated BL patients, 38.2% were in the lowest income quartile compared to 23.6% of HIV-negative BL patients (p < 0.001). Previous studies also demonstrated people living with material deprivation (i.e., those in lower income quartiles) are more likely to participate in risky behavior leading to higher chances of HIV infection [18]. Economic issues leading to homelessness, lack of necessities, and sex exchange often result in nonadherence [18].

The current study evaluated data from 2010 to 2020 and importantly, the COVID-19 pandemic began impacting healthcare in the United States in January of 2020. We were unable to assess the impact of COVID-19 on readmissions during 2020; an ICD-10 diagnosis code for COVID-19 was not available until October 1, 2020. We acknowledge that COVID-19 may have impacted readmission outcomes during 2020 (e.g., readmissions due to COVID-19 infections, shortage of hospital beds available, etc.); however, 90% of our data is pre-COVD-19 (2010–2019). Future research should consider the effect of COVID-19 on hospital admissions and readmissions for the subpopulation of HIV-associated lymphoma patients.

Lastly, the NRD is an administrative database and provides no HIV or BL specific information, particularly treatment status or CD4 count. As such, our study has limitations, particularly regarding lack of CD4 count, AIDS status, opportunistic infections, antiretroviral treatment status and COVID-19 status and vaccination. Relatedly, the NRD only captures diagnoses and procedures identified by ICD-codes thereby limiting the specificity of diagnostic and procedural information. Additionally, the NRD is limited to the specificity of the standardized Healthcare Cost and Utilization Project (HCUP) variables (race, income quartile, expected primary payer); these variables may have less detailed racial and ethnic information compared to patient data. Despite these limitations, we identified 510 HIV-associated BL patients in a national sample. To the best of our knowledge, no previous studies have examined readmission rates and characteristics of HIV-associated BL hospitalizations.

## Conclusion

Our findings suggest that HIV is associated with an increased risk for 30-day readmission in HIV-associated BL. This increased risk of readmission is likely multifactorial and warrants further research to study the effect of chemotherapy regimen, risk of infections and other cancer-specific features related to this outcome.

## Data Availability

The datasets generated during and/or analyzed during the current study are available in the Nationwide Readmissions Database repository, https://hcup-us.ahrq.gov/nrdoverview.jsp.

## List of abbreviations

BL: Burkitt lymphoma
HIV: Human immunodeficiency virus
ART: antiretroviral therapy
NRD: nationwide readmissions database
PWH: people with HIV
